# ﻿A new remarkable representative of Uropodina mites from Seychelles (Acari, Mesostigmata, Trematuridae)

**DOI:** 10.3897/zookeys.1229.142822

**Published:** 2025-02-28

**Authors:** Jenő Kontschán, Sergey G. Ermilov

**Affiliations:** 1 Department of Plant Sciences, Albert Kázmér Faculty of Mosonmagyaróvár, Széchenyi István University, Vár square 2., H-9200 Mosonmagyaróvár, Hungary Széchenyi István University Mosonmagyaróvár Hungary; 2 Institute of Environmental and Agricultural Biology (X-BIO), University of Tyumen, Lenina str. 25, 625000 Tyumen, Russia University of Tyumen Tyumen Russia

**Keywords:** Acari, new combination, new genus, new species, South-East Asia, taxonomy, trematurid mite

## Abstract

A new genus, *Trematirunella***gen. nov.** (Mesostigmata: Uropodina: Trematuridae), with *Trematirunellaseychellia***sp. nov.** as its type species, is described, based on a female, males, and deutonymphs collected in soil samples from the Seychelle Islands. The new genus belongs to the family Trematuridae based on the shape of the corniculi, gnathosomal setae, tritosternum and chelicerae. Members of the new genus bear a preanal suture on the ventral shield, two pairs or more pairs of wide, robust and sword-like setae on the caudal part of the dorsal shield, very dense setation on the caudal area of the marginal shield and an incision on the anterior part of the dorsal shield. These characters are missing in the other genera of Trematuridae. Two previously described species (*Trichouropodalagunae* Hirmatsu & Hirschmann, 1988 and *Trichouropodapalawanensis* Hirschmann & Hirmatsu, 1990) from the Philippines are transferred to the new genus, as *Trematirunellalagunae* (Hirmatsu & Hirschmann, 1988), **comb. nov.** and *Trematirunellapalawanensis* (Hirschmann & Hirmatsu, 1990), **comb. nov.** The new species differs from its congeners in the sculptural pattern of the female genital shield and in the shape of the robust and sword-like setae on the caudal area of the dorsal shield.

## ﻿Introduction

Nabuo Hiramatsu and Werner Hirschmann (1988) described a new, unusual Uropodina species (*Trichouropodalagunae* Hiramatsu & Hirschmann, 1988) from the Philippines and placed it into the large catch-all genus *Trichouropoda* within Hirschmann’s specific systems in the *Trichouropodaelegans*-group. Two years later, Hirschmann and Hiramatsu (1990) described a similar species (*Trichouropodapalawanensis* Hirschmann & Hiramatsu, 1990) again from the Philippines. These species have unique characters (such as the sword-like dorsal setae, dense setation of the marginal shield and the sculptural pattern), but the authors did not mention that the taxonomic position of these species seems to be questionable within both *Trichouropoda* sensu lato and the *Trichouropodaelegans*-group.

During the last decade, the first author spent several weeks in the Natural History Museum of Geneva to study the diversity of tropical Uropodina mites. Among their soil samples, an unusual trematurid mite species was found from the Seychelles, which differs from the other known genera in several unusual characters.

## ﻿Material and methods

Specimens investigated were cleared in lactic acid for a week and afterwards were investigated with a Leica 1000 compound microscope with a drawing tube. Photographs were taken with a Keyence 5000 digital microscope. Specimens examined are stored in 70% ethanol and deposited in the Natural History Museum, Geneva (NHMG). Measurements are given in micrometers (μm). Abbreviations: *st* = sternal setae, *h* = hypostomal setae, *ad* = adanal setae, *p* = poroid, *lf* = lyriform fissure.

## ﻿Taxonomy

### ﻿ Trematuridae

Taxon classificationAnimaliaMesostigmataTrematuridae

Berlese, 1917

EA543FB8-2008-5FD6-9DAD-26306A6E9E6C

Trematurini Berlese, 1917: 9.

#### Diagnosis.

Idiosoma oval, colour reddish-brown or yellowish-brown. Female genital shield scutiform usually with anterior process. Inner margin of marginal shield usually undulate. Corniculi with some (1–4) teeth, internal malae smooth, gnathosmal setae *h1* smooth and often situated on small protuberance, *h2*–*h4* pilose. Base of tritosternum vase-like, with or without lateral spines, tritosternal laciniae laterally pilose, movable digit of chelicerae as long as fixed digit, robust, with 3–5 teeth on both digits. Internal sclerotized node of chelicerae present.

#### Type genus.

*Trematura* Berlese, 1917: 12, by monotypy.

### ﻿ Trematirunella gen. nov.

Taxon classificationAnimaliaMesostigmataTrematuridae

1C7A6914-6CD4-51EB-AB4A-872A402EF8E3

https://zoobank.org/52AB575B-76A7-4E0E-8B58-EB72D5C1EFE6

#### Diagnosis.

Trematurid mites. Idiosoma oval, posteriorly peaked or rounded. Dorsal shield with numerous long and apically pilose or shorter and smooth setae, two or more pairs of setae on caudal area of dorsal shield wide, robust and sword-like. Very dense setation situated on caudal area of marginal shield and an incision visible on anterior part of marginal shield. Ventral shield with preanal suture. Dorsal and ventral idiosoma covered by oval pits. Pedofossae weakly developed.

#### Type species.

*Trematirunellaseychellia* sp. nov.

#### Etymology.

The name of the new genus refers to the family name (Trematuridae).

#### Gender.

Feminine.

#### Remarks.

The new genus differs from the other trematurid genera in the presence of two or more pairs of wide, robust and sword-like setae on the caudal part of the dorsal shield, the very dense setation on the caudal area of the marginal shield and an incision on the anterior part of the marginal shield, which are all missing in the other trematurid genera.

### ﻿Species belonging to the genus

#### ﻿ Trematirunella lagunae

Taxon classificationAnimaliaMesostigmataTrematuridae

(Hiramatsu & Hirschmann, 1988), comb nov.

3ECD6B91-89D3-5946-8E57-13F4028576A6

Trichouropoda lagunae Hiramatsu & Hirschmann, 1988: 194.

##### Occurrence.

Philippines (Hiramatsu and Hirschmann 1988).

#### ﻿ Trematirunella palawanensis

Taxon classificationAnimaliaMesostigmataTrematuridae

(Hirschmann & Hiramatsu, 1990) comb. nov.

8F855377-74B2-5EED-8AF7-CD3F4BFC1705

Trichouropoda palawanensis Hirschmann & Hiramatsu, 1990: 76–81.

##### Occurrence.

Philippines (Hirschmann and Hiramatsu 1990).

#### ﻿ Trematirunella seychellia sp. nov.

Taxon classificationAnimaliaMesostigmataTrematuridae

90FE653D-BE2C-5537-8B0C-999D870BC3C5

https://zoobank.org/BCD646ED-2359-4FB8-8857-33A4EC139D28

**Figure 1.**
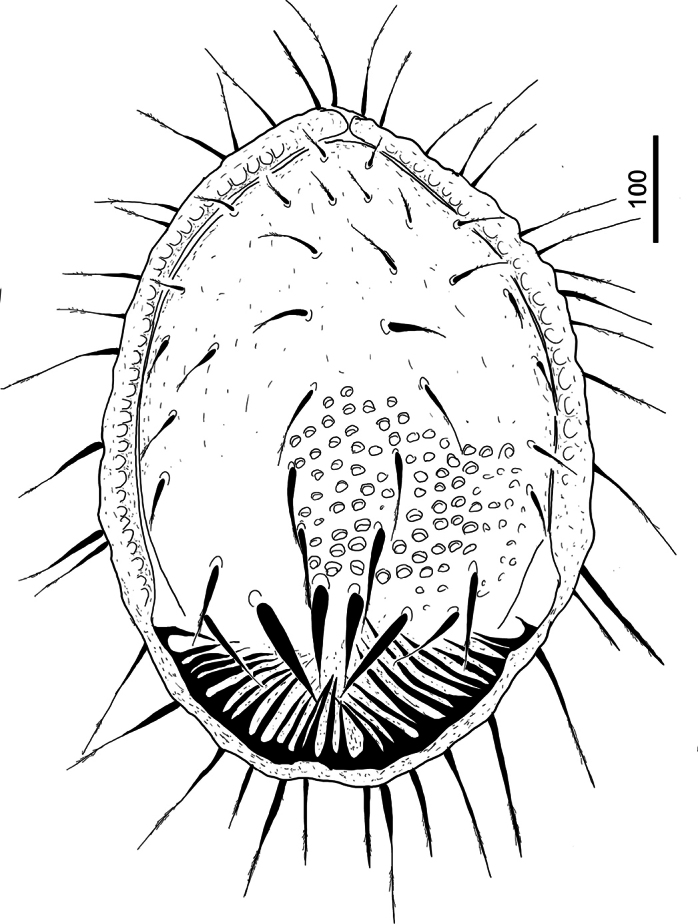
Dorsal view of *Trematirunellaseychellia* gen. nov., sp. nov., holotype, female.

**Figure 2.**
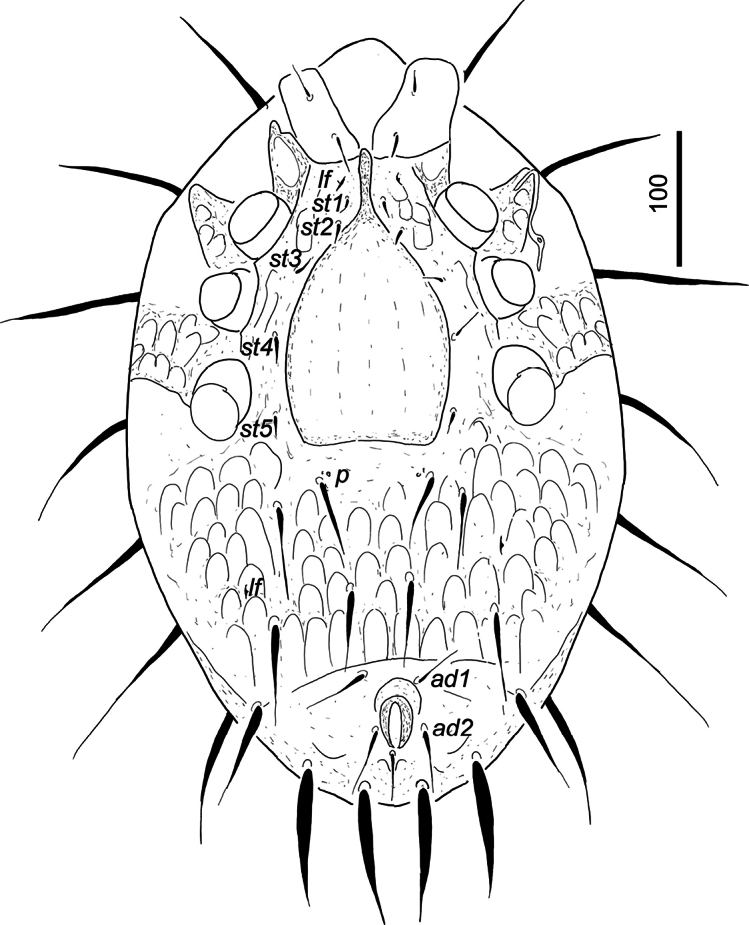
Ventral view of *Trematirunellaseychellia* gen. nov., sp. nov., holotype, female.

**Figure 3.**
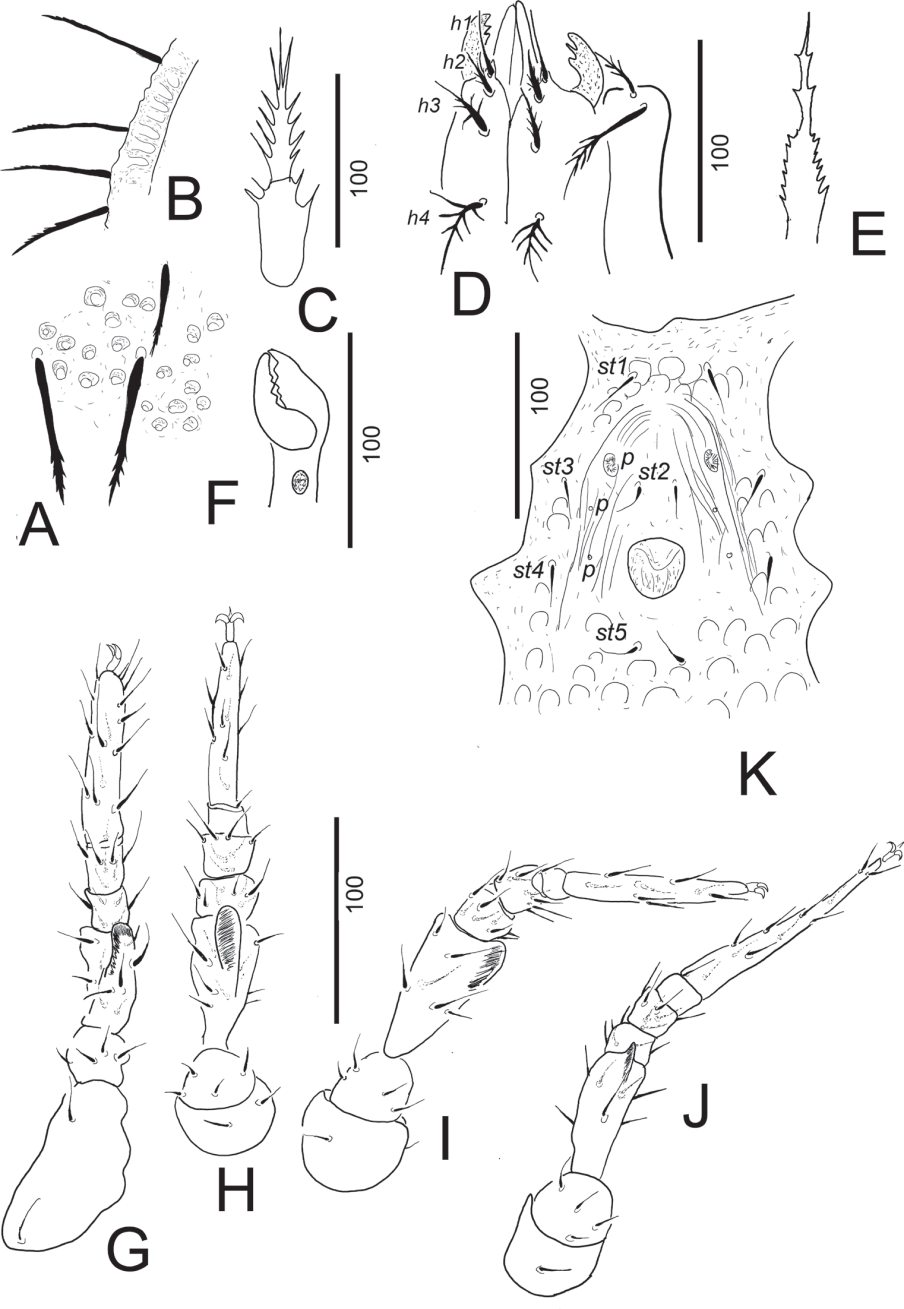
*Trematirunellaseychellia* gen. nov., sp. nov., holotype, female **A** dorsal setae and sculptural pattern **B** marginal setae **C** tritosternum **D** ventral view of gnathosoma **E** epistome **F** lateral view of chelicera **G** leg I, ventral view **H** leg II, ventral view **I** leg III, ventral view **J** leg IV, ventral view **K** intercoxal area of male paratype.

**Figure 4.**
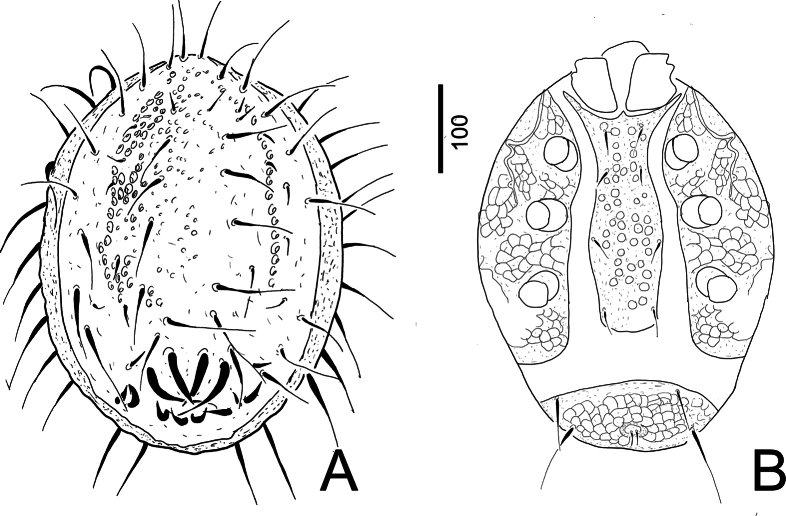
*Trematirunellaseychellia* gen. nov., sp. nov., paratype, deutonymph **A** dorsal view **B** ventral view.

**Figure 5.**
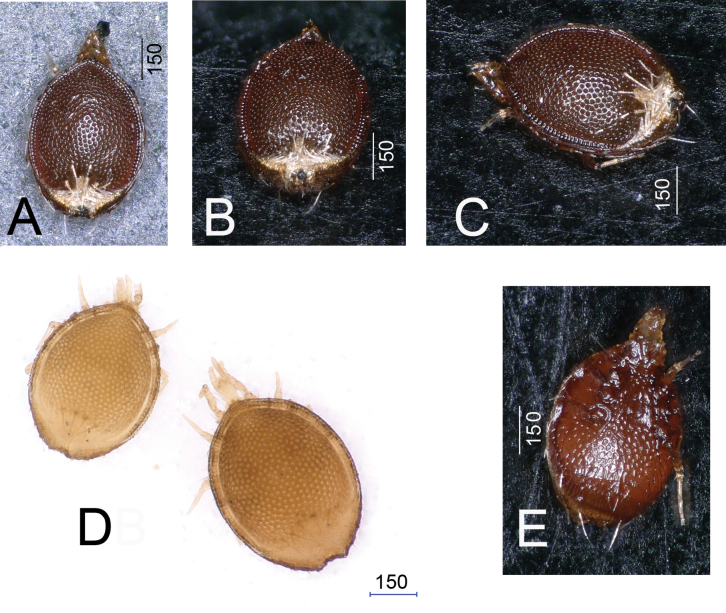
Photos about *Trematirunellaseychellia* gen. nov., sp. nov., paratypes, males **A** dorsal view **B** dorsocaudal view **C** lateral view **D** dorsal view **E** lateroventral view (**A–C**, **E** in dry, **D** in alcohol).

Figs 1 , 2 , 3 , 4 , 5

##### Material examined.

***Holotype*.** • Female. Seychelles, Praslin, 4°20'12"S, 55°44'8"E soil and decaying wood, 27 July 1982, C. Vaucher coll. ***Paratypes*.** Ten males and two deutonymphs. Locality and date same as in holotype.

##### Diagnosis.

Posterior margin of idiosoma rounded. Dorsal setae apically serrate. Setae in row *J* long, reaching basis of next setae. Surface of female genital shield without sculptural pattern. Ventral setae smooth and long.

##### Description.

**Female (N = 1).** Length of idiosoma 635, width at level of coxae IV 462, colour reddish-brown. Shape of idiosoma oval, posterior margin rounded.

***Dorsal idiosoma*** (Figs 1, 5A–D). Marginal and dorsal shields completely separated. Dorsal shield covered by oval pits (*ca* 9–10 × 6–8) with wide court. Dorsal setae apically serrate (Fig. 3A), short (*ca* 33–35) in anterior and lateral areas and long (*ca* 82–97) in central area. Two pairs of robust and sword-like (*ca* 95–102 long) setae situated on caudal area of dorsal shield. Marginal shield with an anterior incision, its inner margin undulate in anterior area. Inner margin of caudal area of marginal shield bearing numerous, long (*ca* 55–105 long) and smooth setae. Marginal setae long (*ca* 82–94) and apically serrate (Fig. 3B).

***Ventral idiosoma*** (Fig. 2). Five pairs of smooth and needle-like (*ca* 10–22 long) sternal setae presented. Setae *st1* inserted at level of anterior margin of coxae II, *st2* at level of mid-coxae II, *st3* at level of anterior margin of coxae III, *st4* at level of posterior margin of coxae III, *st5* at level of posterior margin of coxae IV. Sternal shield with web-like sculptural pattern, one pair of lyriform fissures situated close to *st1*. Preanal suture present. Seven pairs of ventral setae smooth and needle-like, setae anterior to preanal suture shorter (*ca* 55–60) and narrower than setae posterior to preanal suture (*ca* 98–105). Surface of ventral shield ornamented by large oval pits. One pair of poroids situated close to first ventral setae, and one pair of lyriforms fissured on lateral area of ventral shield. Anal opening oval (34 long and 12 wide), anal valves smooth, without euanal setae. Adanal setae (*ad1* and *ad2*) *ca* 36–44 long and needle-like. Postanal seta similar in shape and length to adanal setae. Genital shield scutiform (length 162, basal width 97) with long (*ca* 44) anterior prolongation. Surface of female genital shield without sculptural pattern. Stigmata situated between coxae II and III. Peritremes with hook-shaped prestigmatid part, poststigmatid part short and straight. Pedofossae weakly developed. Tritosternum with vase-like base, with two pairs of apical spines. Its laciniae marginally pilose and apically trifurcated (Fig. 3C).

***Gnathosoma*** (Fig. 3D). Corniculi with three lateral teeth, internal malae narrow and smooth. Hypostomal setae *h1* smooth and needle-like (*ca* 29–32), *h2* (*ca* 21–23), *h3* (*ca* 37–39) and *h4* (*ca* 41–43) marginally serrate. Deutosternal groove narrow, without denticles. Epistome marginally serrate (Fig. 3E), basal part wider than apical part. Chelicerae with internal sclerotized nodes (Fig. 3F). Both digits of chelicerae bearing 3–4 teeth, movable digit as long as fixed digit (*ca* 45–52 long). Both palp trochanter setae pilose, other setae on palp segments smooth.

***Legs*** (Fig. 3G–J). Length of legs (from base of coxae to apex of tarsi): I 280–283, II 245–248, III 270–272, IV 283–284. Leg I with ambulacral claws; all setae smooth, long and need-like. Femora I–IV with ventral flaps.

**Male (N = 10).** Body 630–637 long and 450–460 wide.

***Dorsal idiosoma*.** As in female.

***Ventral idiosoma*** (Figs 3K, 5E). Intercoxal area, with sternal setae and genital shield, as in Fig. 3K. Sternal setae *ca* 23–25 long, smooth and needle-like. Setae *st1* situated at level of mid-coxae II, *st2* and *st3* at level of anterior margin of coxae III, *st4* at level of posterior margin of coxae II, *st5* posterior to genital opening. Surface of sternal shield covered by oval pits, but a U-shaped reticulate sculptural pattern visible anterior and lateral to genital opening. One pair of large poroid-like structure situated between *st2* and *st3* and two small pairs of poroids situated posterior to large ones. Genital shield rounded, but anterior margin a little straight, *ca* 32–34 long and 27–29 wide. Its surface smooth, without eugenital setae and situated between coxae III–IV.

Other characters as in female.

**Deutonymph (N = *2*).** Idiosoma 450–455 long and 346–350 wide.

***Dorsal idiosoma*** (Fig. 4A). Dorsal shield oval, its surface covered by oval pits with wide court. Dorsal setae smooth and apically serrate in different sizes (*ca* 18–72 long). Caudal part of dorsal shield with four pairs of anterior and long (*ca* 85–87 long) and four pairs of posterior and short (*ca* 27–30 long) robust setae. Marginal shield without sculptural pattern and bearing *ca* 72–88 long apically serrate setae.

***Ventral idiosoma*** (Fig. 4B). Sternal shield vase-shaped, covered by oval pits and bearing four pairs of needle-like setae (*ca* 22–24 long). Surface of peritrematal and metapodal shields with web-like sculptural pattern. Prestigmatid part of peritremes long with two bends, postsitgmatid part short and straight. Anal shield wider (203–207) than long (78–82) and bearing one pair of shorter (*ca* 45–47 long) and one pair of longer (*ca* 86–89 long) setae. Surface of anal shield with web-like sculptural pattern. Anal opening wider than long (10–12 × 17–18) and bearing one pair of short (*ca* 16–17) and needle-like setae.

Protonymph and larvae unknown.

##### Etymology.

The name of the new species refers to the islands, where the species was collected.

##### Note.

The most important differences among the known *Trematirunella* species are summarized in Table 1.

**Table 1.** Most important differences among the known *Trematirunella* species.

	*T.lagunae*	*T.palawanensis*	*T.seychellia*
Caudal part of idiosoma	rounded	peaked	rounded
Shape of dorsal setae	smooth	serrate	serrate
Length of dorsal setae	shorter than distance between two setae	longer than distance between two setae	longer distance between two setae
Surface of genital shield	with oval pits	with oval pits	smooth
Length of *st1* and *st2*	two times shorter than *st3*	two times shorter than *st3*	as long as *st3*

##### Zoogeographical note.

*Trematirunella* species have been reported only from the Philippines and the Seychelles. The distance between the two known localities is considerable. Still, there is no information about the occurrence of the members of this genus in some of the most intensively studied countries (like Malaysia, Indonesia, and Thailand) (see Kontschán 2024).

## Supplementary Material

XML Treatment for Trematuridae

XML Treatment for Trematirunella

XML Treatment for Trematirunella lagunae

XML Treatment for Trematirunella palawanensis

XML Treatment for Trematirunella seychellia

## ﻿Additional information

### Author ORCIDs

Jenő Kontschán https://orcid.org/0000-0001-8274-4238

Sergey G. Ermilov https://orcid.org/0000-0002-0913-131X

### Data availability

All of the data that support the findings of this study are available in the main text.
